# Combining Pareto-optimal clusters using supervised learning for identifying co-expressed genes

**DOI:** 10.1186/1471-2105-10-27

**Published:** 2009-01-20

**Authors:** Ujjwal Maulik, Anirban Mukhopadhyay, Sanghamitra Bandyopadhyay

**Affiliations:** 1 Department of Computer Science and Engineering, Jadavpur University, Kolkata – 700032, India; 2 Department of Computer Science and Engineering, University of Kalyani, Kalyani – 741235, India; 3 Machine Intelligence Unit, Indian Statistical Institute, Kolkata – 700108, India

## Abstract

**Background:**

The landscape of biological and biomedical research is being changed rapidly with the invention of microarrays which enables simultaneous view on the transcription levels of a huge number of genes across different experimental conditions or time points. Using microarray data sets, clustering algorithms have been actively utilized in order to identify groups of co-expressed genes. This article poses the problem of fuzzy clustering in microarray data as a multiobjective optimization problem which simultaneously optimizes two internal fuzzy cluster validity indices to yield a set of Pareto-optimal clustering solutions. Each of these clustering solutions possesses some amount of information regarding the clustering structure of the input data. Motivated by this fact, a novel fuzzy majority voting approach is proposed to combine the clustering information from all the solutions in the resultant Pareto-optimal set. This approach first identifies the genes which are assigned to some particular cluster with high membership degree by most of the Pareto-optimal solutions. Using this set of genes as the training set, the remaining genes are classified by a supervised learning algorithm. In this work, we have used a Support Vector Machine (SVM) classifier for this purpose.

**Results:**

The performance of the proposed clustering technique has been demonstrated on five publicly available benchmark microarray data sets, viz., Yeast Sporulation, Yeast Cell Cycle, Arabidopsis Thaliana, Human Fibroblasts Serum and Rat Central Nervous System. Comparative studies of the use of different SVM kernels and several widely used microarray clustering techniques are reported. Moreover, statistical significance tests have been carried out to establish the statistical superiority of the proposed clustering approach. Finally, biological significance tests have been carried out using a web based gene annotation tool to show that the proposed method is able to produce biologically relevant clusters of co-expressed genes.

**Conclusion:**

The proposed clustering method has been shown to perform better than other well-known clustering algorithms in finding clusters of co-expressed genes efficiently. The clusters of genes produced by the proposed technique are also found to be biologically significant, i.e., consist of genes which belong to the same functional groups. This indicates that the proposed clustering method can be used efficiently to identify co-expressed genes in microarray gene expression data.

**Supplementary Website **The pre-processed and normalized data sets, the matlab code and other related materials are available at .

## Background

The progress in the field of microarray technology has made it possible to simultaneously study the expression levels of a large number of genes across different experimental conditions. Microarray technology has applications in the areas of medical diagnosis, bio-medicine, gene expression profiling, etc [1-4]. Usually, the gene expression values during a biological experiment are measured at different time points. A microarray gene expression data, consisting of *g *genes and *h *time points, is typically organized in a 2D matrix *E *= [*e*_*ij*_] of size *g *× *h*. Each element *e*_*ij *_gives the expression level of the *i*th gene at the *j*th time point. Clustering [5], an important microarray analysis tool, is used to identify the sets of genes with similar expression profiles. Clustering methods partition a set of *n *objects into *K *groups based on some similarity/dissimilarity metric where the value of *K *may or may not be known *a priori*. Unlike hard clustering, a fuzzy clustering algorithm produces a *K *× *n *membership matrix *U*(*X*) = [*u*_*kj*_], *k *= 1, ..., *K *and *j *= 1, ..., *n*, where *u*_*kj *_denotes the probability of assigning pattern *x*_*j *_to cluster *C*_*k*_. For probabilistic non-degenerate clustering, 0 <*u*_*kj *_< 1 and $\sum_{k=1}^{K}u_{kj}=1$, 1 ≤ *j *≤ *n *[6].

Genetic algorithms [7] have been effectively used to develop efficient clustering techniques [8,9]. These techniques use a single cluster validity measure as the fitness function to reflect the goodness of an encoded clustering. However, a single cluster validity measure is seldom equally applicable for different kinds of data sets. This article poses the problem of fuzzy partitioning as one of multiobjective optimization (MOO) [10-13]. Unlike single objective optimization, in MOO, search is performed over a number of, often conflicting, objective functions. The final solution set contains a number of Pareto-optimal solutions, none of which can be further improved on any one objective without degrading it in another. A Non-dominated Sorting GA-II (NSGA-II) [13] based multiobjective fuzzy clustering algorithm has been adopted that optimizes the Xie-Beni (XB) index [14] and the fuzzy C-means (FCM) [6] measure (*J*_*m*_) simultaneously [11]. A characteristic of any MOO approach is that it often produces a large number of Pareto-optimal solutions, from which selecting a particular solution is difficult. The existing methods use the characteristics of the Pareto-optimal surface or some external measure for this purpose. However, these approaches almost always pick up one solution from the Pareto-optimal set as the final solution, although evidently all the solutions in this set have some information that is inherently good for the problem in hand. Motivated by this observation, this article describes a novel method to obtain the final solution while considering all the Pareto-optimal solutions by utilizing the input data as a guiding factor. The approach is to integrate the multiobjective clustering technique with a support vector machine (SVM) [15] based classifier to obtain the final solution from the Pareto-optimal set. The procedure involves utilizing the points which are given a high membership degree to a particular class by a majority of the non-dominated solutions. These points are taken as the training points to train the SVM classifier. The remaining points are then classified by the trained SVM classifier to yield the class labels for these points.

Many approaches that solve clustering problems with machine learning algorithms, such as Artificial Neural Networks, Genetic Algorithms, Simulated Annealing etc., can be found in the literature. In [16], an unsupervised self organizing neural network based hierarchical clustering algorithm for gene expression data has been developed. The unsupervised neural network grows adopting the topology of a binary tree. The algorithm combines the advantages of both hierarchical clustering and Self Organizing Map (SOM). In [17], an unsupervised clustering technique based on self-optimizing neural network has been presented. The algorithm is able to find out the most differentiating features for training data and recursively divides them into subgroups. The division of the data is recursively performed till the differences among the subgroups become imperceptible. In [18], a multiple-level hybrid classifier, which combines the supervised decision tree classifiers and unsupervised Bayesian clustering to detect intrusions has been proposed. Clustering using Genetic Algorithms (GA) [8-12] and Simulated Annealing (SA) [19-23] have widely been studied in the literature. The clustering method proposed in this article differs from those mentioned above in the sense that in this algorithm, a novel approach to boost the clustering performance of the multiobjective genetic fuzzy clustering by integrating it with a supervised learning approach is proposed. In this regard, a fuzzy majority voting technique followed by SVM classification is applied on the resultant set of non-dominated solutions in order to obtain the final solution.

The performance of the Multiobjective GA (MOGA) based fuzzy clustering followed by SVM classification (MOGA-SVM) has been demonstrated on five real-life gene expression data sets, viz., Yeast Sporulation, Yeast Cell Cycle, Arabidopsis Thaliana, Human Fibroblasts Serum and Rat CNS data. The superiority of the proposed technique, as compared to MOGA clustering [11], a crisp version of MOGA-SVM, termed as MOGA_*crisp*_-SVM, FCM algorithm [6], single objective GA (SGA) [9], hierarchical average linkage clustering, Self Organizing Map (SOM) clustering [24] and Chinese Restaurant Clustering (CRC) [25], is demonstrated both quantitatively and visually. The use of different SVM kernels has been explored. The superiority of the MOGA-SVM clustering technique has been proved to be statistically significant through statistical tests. Finally a biological significance test has been conducted to establish that the proposed technique produces functionally enriched clusters.

## Results and Discussion

The performance of the proposed MOGA-SVM clustering has been evaluated on five publicly available real life gene expression data sets, *viz*., Yeast Sporulation, Yeast Cell Cycle, Arabidopsis Thaliana, Human Fibroblasts Serum and Rat CNS data. First, the effect of the parameter *β *(majority voting threshold) on the performance of MOGA-SVM clustering has been examined. Thereafter, we examined the use of different kernel functions and compared their performances. The performance of the proposed technique has also been compared with those of fuzzy MOGA clustering (without SVM) [10,11], FCM [6], single objective genetic clustering scheme which minimizes XB validity measure (SGA) [9], average linkage method [26], SOM [24] and CRC [25]. Moreover, a crisp version of MOGA-SVM clustering (MOGA_*crisp*_-SVM) is considered for comparison in order to establish the utility of incorporating fuzziness. Unlike fuzzy MOGA-SVM, which uses the FCM based chromosome update, in MOGA_*crisp*_-SVM, chromosomes are updated using the *K*-means like center update process and the crisp versions of *J*_*m *_and *XB *indices are optimized simultaneously. To obtain the final clustering solution from the set of non-dominated solutions, similar procedure as in fuzzy MOGA-SVM is followed. Note that in the case of MOGA_*crisp*_-SVM, as membership degrees are either 0 or 1, hence the membership threshold parameter *α *is not required. The statistical and biological significance of the clustering results have also been evaluated.

### Effect of Majority Voting Threshold *β*

**Figure 1:**
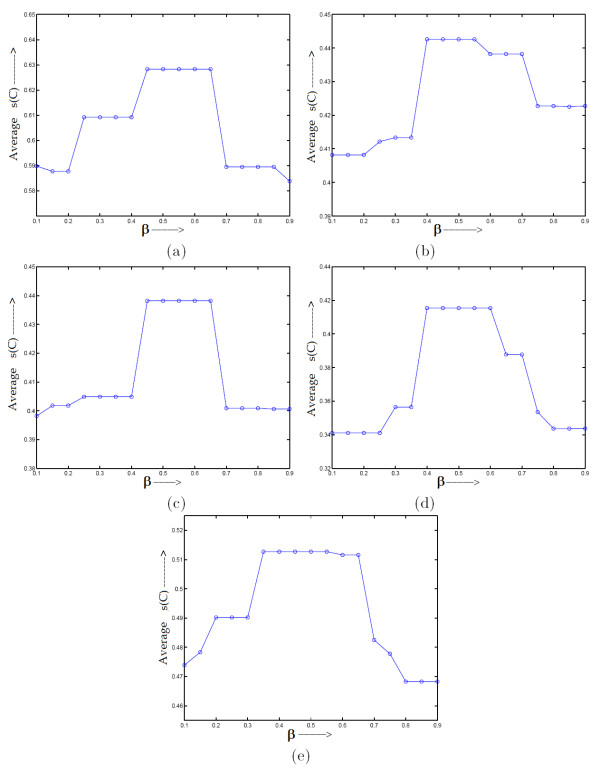
**Variation of average *s*(*C*) index values produced by MOGA-SVM (RBF) clustering over different *β *values ranging from 0.1 to 0.9 for the data sets (a) Sporulation, (b) Cell Cycle, (c) Arabidopsis, (d) Serum, (e) Rat CNS**.

In this section we have analyzed how the parameter *β *(majority voting threshold) affects the performance of the proposed MOGA-SVM clustering technique. The algorithm has been executed for a range of *β *values starting from 0.1 to 0.9 with a step size of 0.05 for all the data sets. The results reported in this section are for the Radial Basis Function (RBF) [15,27]. Experiments with other kernel functions are also found to provide similar behavior. For each value of *β*, the average value of the silhouette index (*s*(*C*)) scores over 20 runs has been considered. The parameter *α *(membership threshold) has been kept constant at 0.5. The variation of average *s*(*C*) scores for different values of *β *are demonstrated in Fig. 1 for the five data sets.

It is evident from Fig. 1 that for all the data sets, MOGA-SVM behaves similarly in terms of variation of average *s*(*C*) over the range of *β *values. The general trend is that first the average *s*(*C*) scores get improved with increasing *β *value, then remains almost constant in the range of around 0.4 to 0.6, and then deteriorates with further increase in *β *value. This behavior is quite expected, as for small value of *β*, the training set will contain lot of low-confidence points, which causes the class boundaries to be defined incorrectly for SVM. On the other hand, when *β *value is very high, the training set is small and contains only a few high confidence points. Thus the hyperplanes between the classes cannot be properly defined. In some range of *β *(around 0.4 to 0.6), a tradeoff is obtained between the size of the training set and its confidence level. Hence in this range, MOGA-SVM provides the best *s*(*C*) index scores. With this observation, in all the experiments hereafter, *β *value has been kept constant at 0.5.

### Performance of MOGA-SVM for Different Kernels

Four kernel functions, viz., linear, polynomial, sigmoidal and RBF are considered in this article. In this section, a study has been made on how the different kernel functions perform for the five data sets. Table 1 reports the *s*(*C*) scores (averaged over 20 runs) produced by MOGA-SVM with the four different kernel functions for the five data sets. The average *s*(*C*) scores provided by MOGA (without SVM) over 20 runs is also reported for each data set. Moreover, the number of clusters *K *(corresponding to the solution providing the best silhouette index score) found for the different data sets has been shown.

**Table 1:** Average Silhouette index scores over 20 runs of MOGA-SVM with different kernel functions for the five gene expression data sets along with the average Silhouette index score of the MOGA (without SVM)

Algorithm	Sporulation	Cell Cycle	Arabidopsis	Serum	Rat CNS
	
	*K *= 6	*K *= 5	*K *= 4	*K *= 6	*K *= 6
MOGA-SVM (linear)	0.5852	0.4398	0.4092	0.4017	0.4966
MOGA-SVM (polynomial)	0.5877	0.4127	0.4202	0.4112	0.5082
MOGA-SVM (sigmoidal)	0.5982	0.4402	0.4122	0.4112	0.5106
MOGA-SVM (RBF)	0.6283	0.4426	0.4312	0.4154	0.5127
MOGA (without SVM)	0.5794	0.4392	0.4011	0.3947	0.4872

As is evident from the table, irrespective of the kernel function considered, use of SVM provides better *s*(*C*) score compared to the MOGA(without SVM). This is expected since the MOGA-SVM techniques provide equal importance to all the non-dominated solutions, rather than a single one. Thus through fuzzy voting, the core group of genes for each cluster is identified and the class labels of the remaining genes are predicted by the SVM. It can also be noticed from the table that the silhouette index produced by the RBF kernel is greater than those produced by the other kernels. This is because RBF kernels are known to perform well in case of spherical shaped clusters, which is very common in case of gene expression data sets. Henceforth, MOGA-SVM will indicate MOGA-SVM with RBF kernel only.

### Comparative Results

Table 2 reports the average *s*(*C*) index values provided by MOGA-SVM (RBF), MOGA (without SVM), MOGA_*crisp*_-SVM (RBF), FCM, SGA, Average linkage, SOM and CRC clustering over 20 runs of the algorithms for the five real life data sets considered here. Also the number of clusters *K *obtained corresponding to the maximum *s*(*C*) index score for each algorithm is reported. The values reported in the tables show that for all the data sets, MOGA-SVM provides the best *s*(*C*) index score. MOGA_*crisp*_-SVM (RBF) also provides reasonably good *s*(*C*) index scores, but is outperformed by MOGA-SVM for all the data sets. This indicates the utility of incorporating fuzziness in MOGA clustering. Interestingly, while incorporation of SVM based training improves the performance of MOGA clustering, the latter also provides, in most cases, better *s*(*C*) values than SGA and the other non-genetic approaches. Only for Yeast Sporulation and Arabidopsis Thaliana data sets, the results for MOGA (without SVM) are slightly inferior to those of SOM and CRC, respectively. However, the performance of the proposed MOGA-SVM is the best for all the data sets.

**Table 2:** Average Silhouette index scores over 20 runs of different algorithms for the five gene expression data sets

Algorithm	Sporulation		Cell Cycle		Thaliana		Serum		Rat CNS	
	
	*K*	*s*(*C*)	*K*	*s*(*C*)	*K*	*s*(*C*)	*K*	*s*(*C*)	*K*	*s*(*C*)
MOGA-SVM (RBF)	6	0.6283	5	0.4426	4	0.4312	6	0.4154	6	0.5127
MOGA (without SVM)	6	0.5794	5	0.4392	4	0.4011	6	0.3947	6	0.4872
MOGA_*crisp*_-SVM (RBF)	6	0.5971	5	0.4271	4	0.4187	6	0.3908	6	0.4917
FCM	7	0.4755	6	0.3872	4	0.3642	8	0.2995	5	0.4050
SGA	6	0.5703	5	0.4221	4	0.3831	6	0.3443	6	0.4486
Average linkage	6	0.5007	4	0.4388	5	0.3151	4	0.3562	6	0.4122
SOM	6	0.5845	6	0.3682	5	0.2133	6	0.3235	5	0.4430
CRC	8	0.5622	5	0.4288	4	0.4109	10	0.3174	4	0.4423

MOGA has determined 6, 5, 4, 6 and 6 number of clusters for the Sporulation, Cell Cycle, Arabidopsis, Serum and Rat CNS data sets, respectively. This conforms to the findings in the literature [28-31]. Hence it is evident from the table that while MOGA (without SVM) and MOGA_*crisp*_-SVM (RBF) are generally superior to the other methods, MOGA-SVM is the best among all the competing methods for all the data sets considered here.

**Figure 2:**
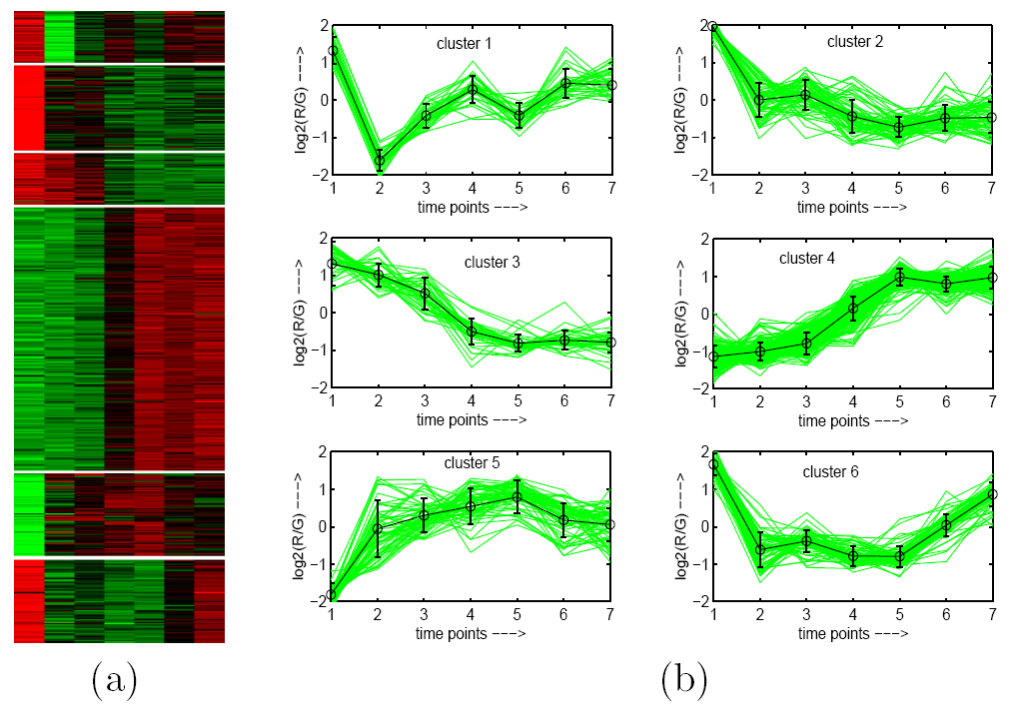
**Yeast Sporulation data clustered using MOGA-SVM clustering method**. (a) Eisen plot, (b) Cluster profile plots.

**Figure 3:**
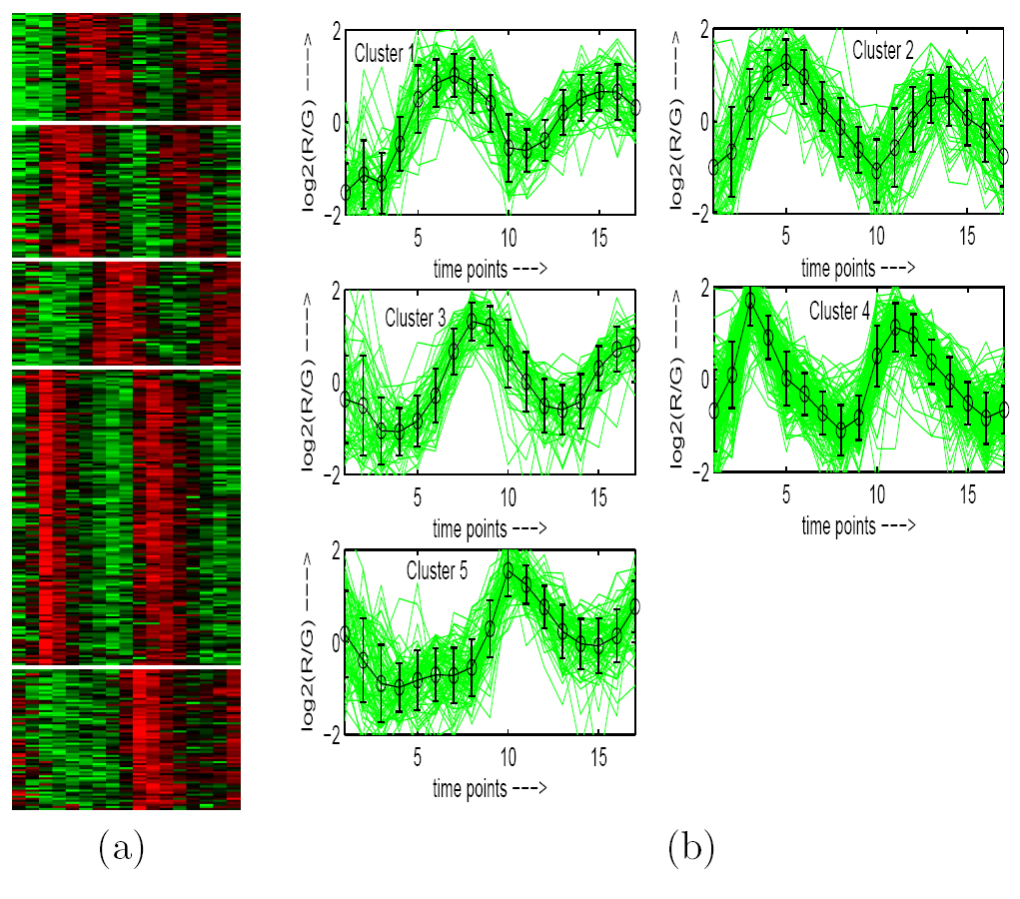
**Yeast Cell Cycle data clustered using MOGA-SVM clustering method**. (a) Eisen plot, (b) Cluster profile plots.

**Figure 4:**
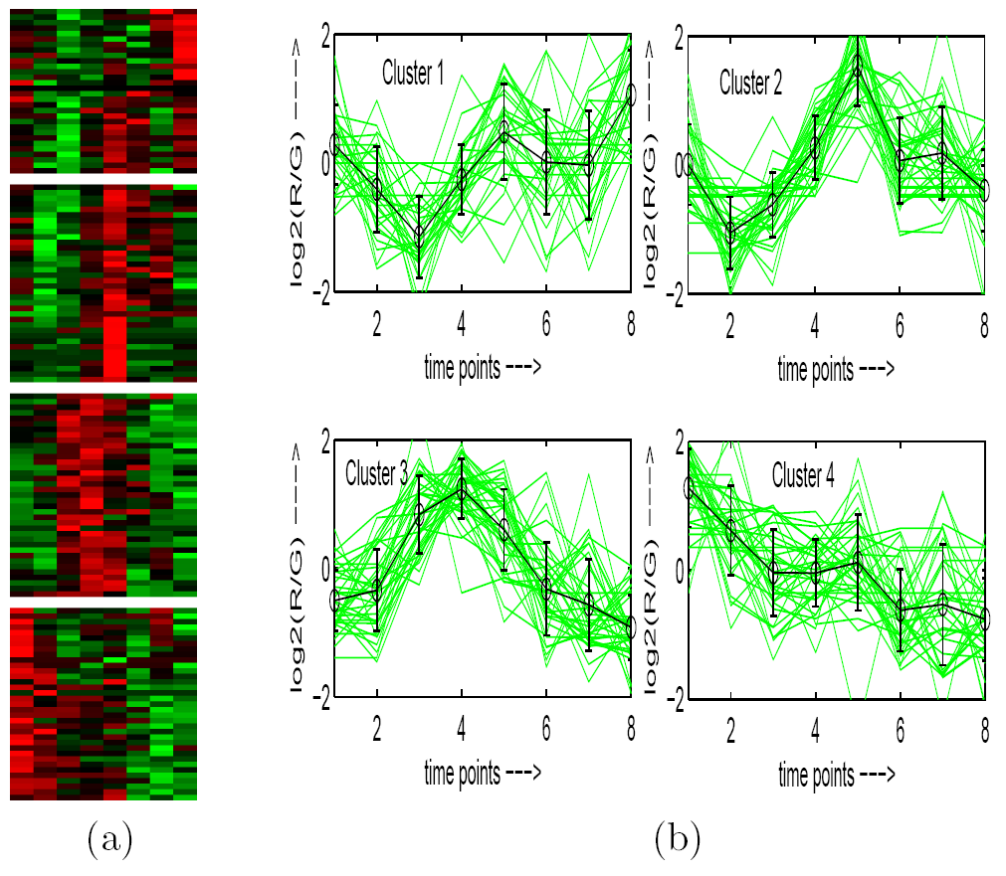
**Arabidopsis Thaliana data clustered using MOGA-SVM clustering method**. (a) Eisen plot, (b) Cluster profile plots.

**Figure 5:**
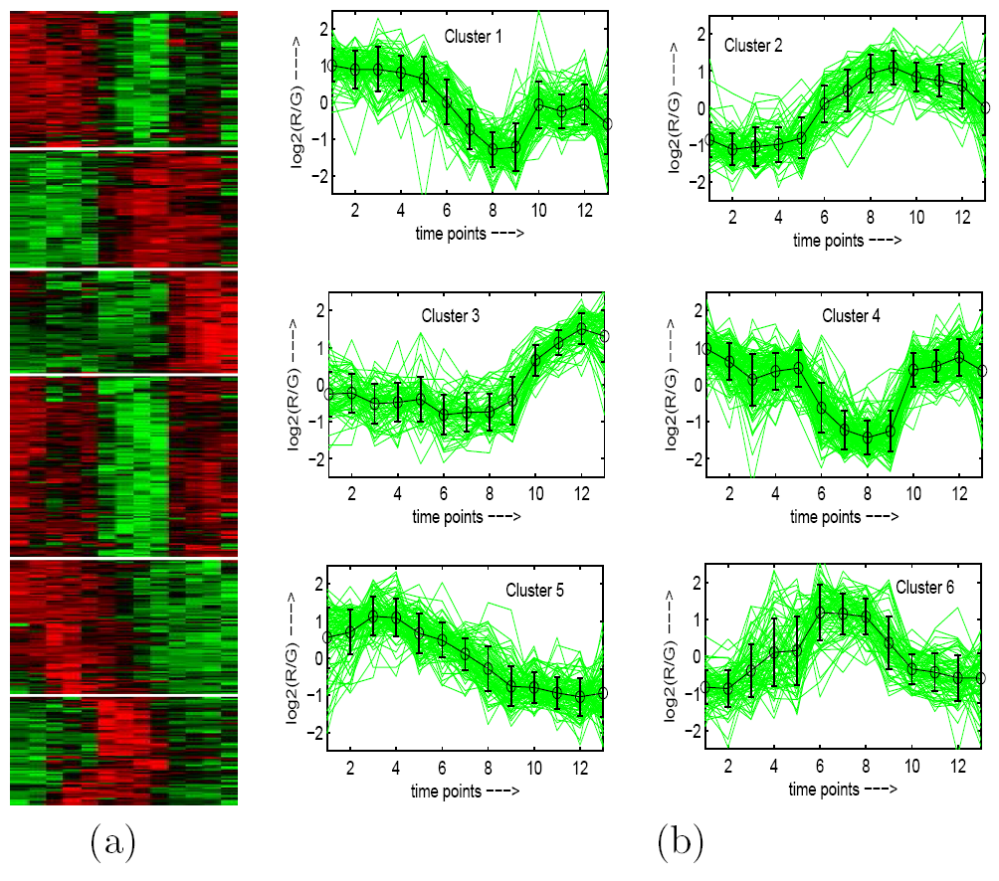
**Human Fibroblasts Serum data clustered using MOGA-SVM clustering method**. (a) Eisen plot, (b) Cluster profile plots.

**Figure 6:**
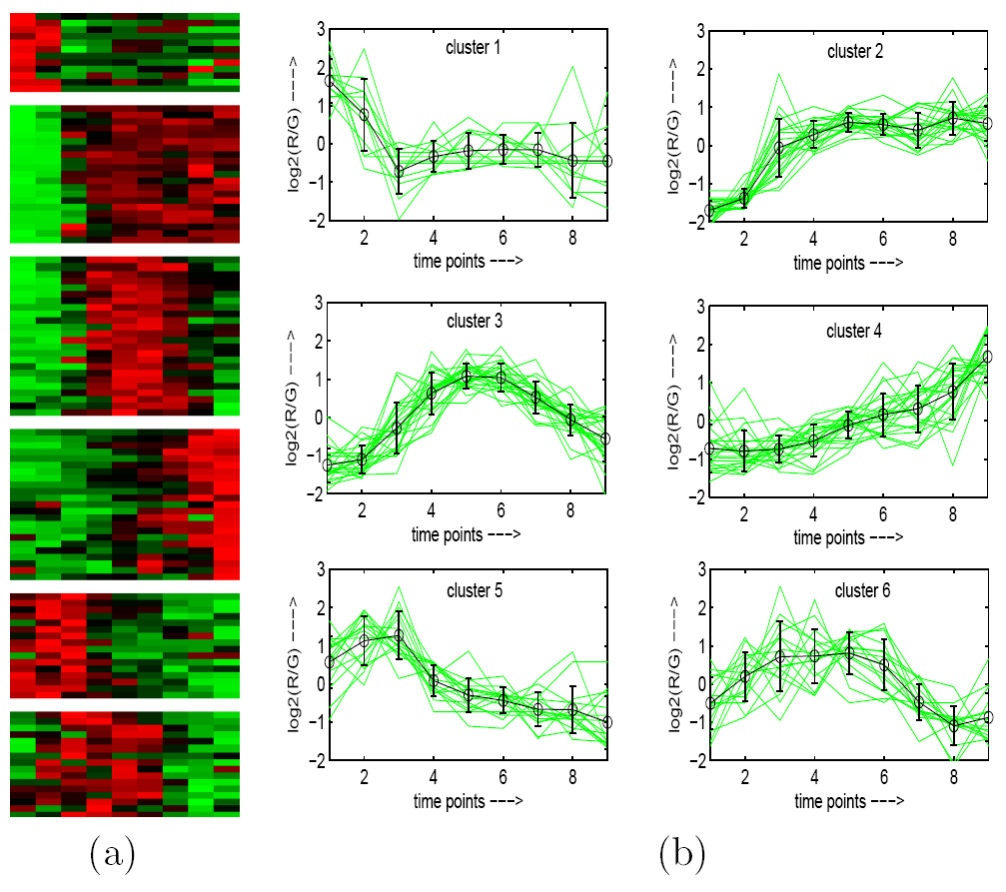
**Rat CNS data clustered using MOGA-SVM clustering method**. (a) Eisen plot, (b) Cluster profile plots.

To demonstrate visually the result of MOGA-SVM clustering, Figs. 2, 3, 4, 5, 6 show the Eisen plot and cluster profile plots provided by MOGA-SVM on the five data sets, respectively. For example, the 6 clusters of the Yeast Sporulation data are very prominent as shown in the Eisen plot (Fig. 2(a)). It is evident from the figure that the expression profiles of the genes of a cluster are similar to each other and they produce similar color patterns. The cluster profile plots (Fig. 2(b)) also demonstrate how the expression profiles for the different groups of genes differ from each other, while the profiles within a group are reasonably similar. Similar results are obtained for the other data sets also.

The proposed technique performs better compared to the other clustering methods mainly because of the following reasons: first of all, this is a multiobjective clustering method. Simultaneous optimization of multiple cluster validity measures helps to cope with different characteristics of the partitioning and leads to higher quality solutions and an improved robustness towards the different data properties. Secondly, the strength of supervised learning has been integrated with the multiobjective clustering efficiently. As each of the solutions in the final non-dominated set contains some information about the clustering structure of the data set, combining them with the help of majority voting followed by supervised classification yields a high quality clustering solution. Finally, incorporation of fuzziness makes the proposed technique better equipped in handling overlapping clusters.

### Statistical Significance Test

To establish that MOGA-SVM is significantly superior compared to the other algorithms, a non-parametric statistical significance test called Wilcoxon's rank sum test for independent samples [32] has been conducted at the 5% significance level. Except from Average linkage, all other methods considered here are probabilistic in nature, i.e., they may produce different clustering results in different runs depending on the initialization. It has been found that in all the runs, MOGA-SVM produces better *s*(*C*) index scores compared to those produced by Average linkage algorithm. Therefore, the Average linkage algorithm is not considered in the statistical test conducted. Seven groups, corresponding to the seven algorithms (1. MOGA-SVM (RBF), 2. MOGA (without SVM), 3. MOGA_*crisp*_-SVM (RBF), 4. FCM, 5. SGA, 6. SOM, 7. CRC), have been created for each data set. Each group consists of the *s*(*C*) index scores produced over 20 runs of the corresponding algorithm. The median values of each group for all the data sets are reported in Table 3.

**Table 3:** Median values of Silhouette index scores over 20 consecutive runs of different algorithms.

Algorithm	Sporulation	Cell Cycle	Arabidopsis	Serum	Rat CNS
MOGA-SVM (RBF)	0.6288	0.4498	0.4329	0.4148	0.5108
MOGA (without SVM)	0.5766	0.4221	0.4024	0.3844	0.4822
MOGA_*crisp*_-SVM (RBF)	0.6002	0.4301	0.4192	0.3901	0.4961
FCM	0.4686	0.3812	0.3656	0.3152	0.4113
SGA	0.5698	0.4315	0.3837	0.3672	0.4563
Average linkage	0.5007	0.4388	0.3151	0.3562	0.4122
SOM	0.5786	0.3823	0.2334	0.3352	0.4340
CRC	0.5619	0.4271	0.3955	0.3246	0.4561

As is evident from Table 3, the median values of *s*(*C*) scores for MOGA-SVM are better than those for the other algorithms. To establish that this goodness is statistically significant, Table 4 reports the *p-values *produced by Wilcoxon's rank sum test for comparison of two groups (group corresponding to MOGA-SVM and a group corresponding to some other algorithm) at a time. As a null hypothesis, it is assumed that there are no significant difference between the median values of two groups. Whereas, the alternative hypothesis is that there is significant difference in the median values of the two groups. All the *p-values *reported in the table are less than 0.05 (5% significance level). This is strong evidence against the null hypothesis, indicating that the better median values of the performance metric produced by MOGA-SVM is statistically significant and has not occurred by chance.

**Table 4:** *p-values *produced by Wilcoxon's rank sum test comparing MOGA-SVM with other algorithms.

Data Sets	p-values (comparing median values of Silhouette index of MOGA-SVM with other algorithms)					
	
	MOGA (without SVM)	FCM	MOGA_*crisp*_-SVM	SGA	SOM	CRC
Sporulation	2.10E-03	2.17E-05	1.32E-03	2.41E-03	11.5E-03	5.20E-03
Cell Cycle	2.21E-03	1.67E-05	2.90E-05	1.30E-04	1.44E-04	1.90E-04
Arabidopsis	1.62E-03	1.43E-04	1.78E-03	5.80E-05	2.10E-03	1.08E-05
Serum	1.30E-04	1.52E-04	3.34E-04	1.48E-04	1.44E-04	1.39E-04
Rat CNS	1.53E-04	1.08E-05	2.10E-04	1.53E-04	1.43E-04	1.68E-04

### Biological Significance

The biological relevance of a cluster can be verified based on the statistically significant Gene Ontology (GO) annotation database . This is used to test the functional enrichment of a group of genes in terms of three structured, controlled vocabularies (ontologies), *viz*., associated biological processes, molecular functions and biological components. The degree of functional enrichment (*p-value*) is computed using a cumulative hypergeometric distribution. This measures the probability of finding the number of genes involved in a given GO term (i.e., function, process, component) within a cluster. From a given GO category, the probability *p *of getting *k *or more genes within a cluster of size *n*, can be defined as [33]:

(1) $$p=1-\sum_{i=0}^{k-1}\frac{\left(\begin{array}{c} f\\ i \end{array}\right)\left(\begin{array}{c} g-f\\ n-i \end{array}\right)}{\left(\begin{array}{c} g\\ n \end{array}\right)},$$

where *f *and *g *denote the total number of genes within a category and within the genome, respectively. Statistical significance is evaluated for the genes in a cluster by computing the *p-value *for each GO category. This signifies how well the genes in the cluster match with the different GO categories. If the majority of genes in a cluster have the same biological function, then it is unlikely that this takes place by chance and the *p-value *of the category will be close to 0.

**Figure 7:**
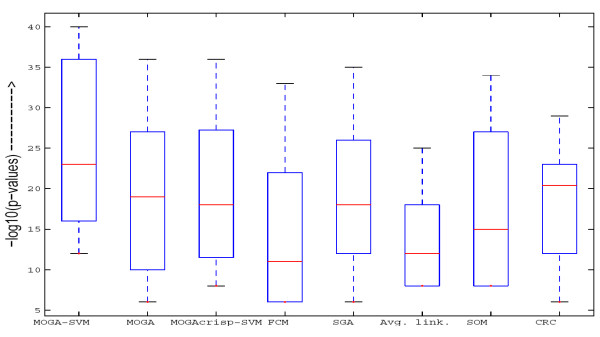
**Boxplots of the *p-values *of the most significant GO terms of all the clusters having at least one significant GO term as obtained by different algorithms for Yeast Sporulation data**. The *p-values *are log-transformed for better readability.

The biological significance test for Yeast Sporulation data has been conducted at the 1% significance level. For different algorithms, the number of clusters for which the most significant GO terms have a *p-value *less than 0.01 (1% significance level) are as follows: MOGA-SVM – 6, MOGA (without SVM) – 6, MOGA_*crisp*_-SVM (RBF) – 6, FCM – 4, SGA – 6, Average linkage – 4, SOM – 4 and CRC – 6. In Fig. 7, the boxplots of the *p-values *of the most significant GO terms of all the clusters having at least one significant GO term as obtained by the different algorithms are shown. The *p-values *are log-transformed for better readability. It is evident from the figure that the boxplot corresponding to MOGA-SVM method has lower *p-values *(i.e., higher -log_10 _(*p-value*)). This indicates that the clusters identified by MOGA-SVM are more biologically significant and functionally enriched compared to the other algorithms.

As an illustration, Table 5 reports the three most significant GO terms (along with the corresponding *p-values*) shared by the genes of each of the 6 clusters identified by MOGA-SVM technique (Fig. 2). As is evident from the table, all the clusters produced by MOGA-SVM clustering scheme are significantly enriched with some GO categories, since all the *p-values *are less than 0.01 (1% significance level). This establishes that the proposed MOGA-SVM clustering scheme is able to produce biologically relevant and functionally enriched clusters.

**Table 5:** The three most significant GO terms and the corresponding *p-values *for each of the 6 clusters of Yeast Sporulation data as found by MOGA-SVM clustering technique

Clusters	Significant GO term	*p-value*
Cluster 1	ribosome biogenesis and assembly – GO:0042254	1.4E-37
	intracellular non-membrane-bound organelle – GO:0043232	1.38E-23
	organelle lumen – GO:0043233	9.46E-21

Cluster 2	nucleotide metabolic process – GO:0009117	1.32E-8
	glucose catabolic process – GO:0006007	2.86E-4
	external encapsulating structure – GO:0030312	3.39E-4

Cluster 3	organic acid metabolic process – GO:0006082	1.86E-14
	amino acid and derivative metabolic process – GO:0006519	4.35E-4
	external encapsulating structure – GO:0030312	6.70E-4

Cluster 4	spore wall assembly (sensu Fungi) – GO:0030476	8.97E-18
	sporulation – GO:0030435	2.02E-18
	cell division – GO:0051301	7.92E-16

Cluster 5	M phase of meiotic cell cycle – GO:0051327	1.71E-23
	M phase – GO:0000279	1.28E-20
	meiosis I – GO:0007127	5.10E-22

Cluster 6	cytosolic part – GO:0044445	1.4E-30
	cytosol – GO:0005829	1.4E-30
	ribosomal large subunit assembly and maintenance – GO:0000027	7.42E-8

## Conclusion

This article proposes a novel method for obtaining a final solution from the set of non-dominated solutions produced by an NSGA-II based real-coded multiobjective fuzzy clustering scheme, that optimizes Xie-Beni (*XB*) index and the *J*_*m *_simultaneously. In this regard, a fuzzy voting technique followed by support vector machine based classification has been utilized. Results on five real-life gene expression data sets have been demonstrated. Use of different kernel methods is investigated whence the RBF kernel is found to perform the best.

The performance of the proposed technique has been compared with those of MOGA (without SVM), MOGA_*crisp*_-SVM (RBF), FCM, SGA, Average linkage, SOM and CRC clustering methods. The results have been demonstrated both quantitatively and visually using cluster visualization tools. The proposed MOGA-SVM clustering technique consistently outperformed the other algorithms considered here as it integrates multiobjective optimization, fuzzy clustering and supervised learning in an effective manner. Statistical superiority has been established through statistical significance tests. Moreover biological significance tests have been conducted to establish that the clusters identified by the proposed technique are biologically significant.

As a scope of further research, performance of other MOGA techniques, such as AMOSA [23] is to be tested. Also, combination of MOGA clustering with different popular supervised classification tools other than SVM can also be studied.

## Methods

### Multiobjective Optimization

The multiobjective optimization can formally be stated as [34]: Find the vector ${\bar{x}}^{*}={\left[x_{1}^{*},x_{2}^{*},...,x_{n}^{*}\right]}^{T}$ of decision variables which satisfies a number of equality and inequality constraints and optimizes the vector function $\bar{f}(\bar{x})={\left[f_{1}(\bar{x}),f_{2}(\bar{x}),...,f_{k}(\bar{x})\right]}^{T}$. The constraints define the feasible region F which contains all the admissible solutions. Any solution outside this region is inadmissible since it violates one or more constraints. The vector ${\bar{x}}^{*}$ denotes an optimal solution in F. The concept of *Pareto optimality *is useful in the domain of multiobjective optimization. A formal definition of Pareto optimality from the viewpoint of the minimization problem may be given as follows: A decision vector ${\bar{x}}^{*}$ is called Pareto-optimal if and only if there is no $\bar{x}$ that dominates ${\bar{x}}^{*}$, i.e., there is no $\bar{x}$ such that ∀*i *∈ {1, 2, ..., *k*}, $f_{i}(\bar{x})\leq f_{i}({\bar{x}}^{*})$ and ∃*i *∈ {1, 2, ..., *k*}, $f_{i}(\bar{x})<f_{i}({\bar{x}}^{*})$. In words, ${\bar{x}}^{*}$ is Pareto-optimal if there exists no feasible vector $\bar{x}$ which causes a reduction on some criterion without a simultaneous increase in at least one other. In general, Pareto optimality usually admits a set of solutions called *non-dominated *solutions.

There are a number of multiobjective optimization techniques available. Among them, the GA based techniques such as NSGA-II [13], SPEA and SPEA2 [35] are very popular. The multiobjective fuzzy clustering scheme [11] considered here uses NSGA-II as an underlying multiobjective framework for developing the proposed fuzzy clustering algorithm.

### Multiobjective Fuzzy Clustering

This section briefly describes the NSGA-II based multiobjective fuzzy clustering scheme (MOGA) [11]. The algorithm MOGA uses real valued chromosomes that denote the co-ordinates of the cluster centers and each has length *K *× *d*, where *K *is the number of clusters and *d *is dimension of the data. Each chromosome in the initial population consists of the co-ordinates of *K *random points from the data set. Two cluster validity indices, Xie-Beni (*XB*) [14] and fuzzy C-means (FCM) measure (*J*_*m*_) [6] are simultaneously optimized. For computing the objective functions, first the centers *V *= {*v*_1_, *v*_2_, ..., *v*_*K*_} encoded in a given chromosome are extracted. The fuzzy membership values *u*_*ik*_, *i *= 1, 2, ..., *K*, *k *= 1, 2, ..., *n *are computed using the following equation [6]:

(2) $$\begin{array}{cccc} u_{ik}=\frac{1}{\sum_{j=1}^{K}{\left(\frac{D(v_{i},x_{k})}{D(v_{j},x_{k})}\right)}^{\frac{2}{m-1}}}, & \text{for} & 1\leq i\leq K; & 1\leq k\leq n, \end{array}$$

where *D*(*v*_*i*_, *x*_*k*_) denotes the distance between *i*th cluster center and *k*th data point and *m *∈ {1, ∞} is the fuzzy exponent. In this article, the Correlation based distance measure is used. Subsequently each cluster center *v*_*i*_, *i *= 1, 2, ..., *K*, is updated using the following equation [6]:

(3) $$\begin{array}{cc} v_{i}=\frac{\sum_{k=1}^{n}{\left(u_{ik}\right)}^{m}x_{k}}{\sum_{k=1}^{n}{\left(u_{ik}\right)}^{m}}, & 1\leq i\leq K. \end{array}$$

The membership values are then recomputed using Eq. (2). The *XB *index is defined as a function of the ratio of the total variation *σ *to the minimum separation *sep *of the clusters. Here *σ *and *sep *can be written as:

(4) $$\sigma (U,V;X)=\sum_{i=1}^{K}\sum_{k=1}^{n}u_{ik}^{2}D^{2}(v_{i},x_{k}),$$

and

(5) $$sep(V)=\underset{i\neq j}{\min}\{D^{2}(v_{i},v_{j})\}.$$

The *XB *index is then written as [14]:

(6) $$XB(U,V;X)=\frac{\sigma (U,V;X)}{n\times sep(V)}.$$

Note that when the partitioning is compact and the clusters are well separated, the value of *σ *should be low while *sep *should be high, thereby yielding lower values of the *XB *index. The objective is therefore to minimize it.

The other objective is the *J*_*m *_measure optimized by the FCM algorithm. This computes the global fuzzy variance of the clusters and this is expressed by the following equation [6]:

(7) $$J_{m}=\sum_{j=1}^{n}\sum_{k=1}^{K}u_{kj}^{m}D^{2}(v_{k},x_{j}).$$

**Figure 8:**
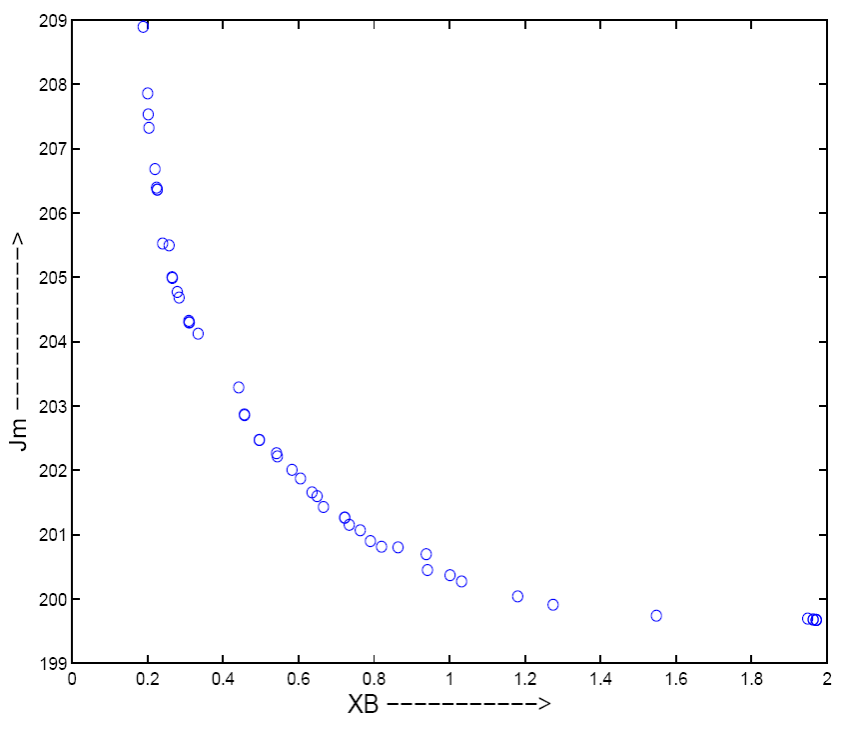
**The final non-dominated Pareto-optimal front obtained by MOGA clustering for Yeast Sporulation data set**.

*J*_*m *_is to be minimized to get compact clusters. *XB *and *J*_*m *_indices are to an extent contradictory in nature. *XB *index is responsible for both compactness and separation for the clusters, whereas *J*_*m *_only represents the global compactness of the clusters. For the purpose of illustration, Fig. 8 shows the Pareto front obtained by the multiobjective fuzzy clustering for Yeast Sporulation data set. The Pareto front indicates that the two objective functions are in conflict with each other.

Crowded binary tournament selection [13] followed by conventional crossover and mutation operators is used here. NSGA-II uses the elitist model where the non-dominated solutions of the parent and child populations are propagated to the next generation in order to keep track of the best solutions obtained so far. The algorithm has been executed for a fixed number of generations. It produces a set of non-dominated solutions in the last generation.

### Support Vector Machine

Support vector machine (SVM) classifiers are inspired by statistical learning theory and they perform structural risk minimization on a nested set structure of separating hyperplanes [15,27]. Fundamentally the SVM classifier is designed for two-class problems. Viewing the input data as two sets of vectors in a *p*-dimensional space, an SVM constructs a separating hyperplane in that space, the one which maximizes the margin between the two classes of points. To compute the margin, two parallel hyperplanes are constructed on each side of the separating one, which are "pushed up against" the two classes of points. Intuitively, a good separation is achieved by the hyperplane that has the largest distance to the neighboring data points of both the classes. The larger the margin or distance between these parallel hyperplanes, the better is the generalization error of the classifier. It can be extended to handle multi-class problems by designing a number of one-against-all or one-against-one two-class SVMs.

Kernel functions are used for mapping the input space to a higher dimensional feature space so that the classes become linearly separable. Use of four popular kernel functions has been studied in this article. These are:

Linear: *K*(*x*_*i*_, *x*_*j*_) = $x_{i}^{T}x_{j}$

Polynomial: *K*(*x*_*i*_, *x*_*j*_) = ${\left(\gamma x_{i}^{T}x_{j}+r\right)}^{d}$

Sigmoidal: *K*(*x*_*i*_, *x*_*j*_) = $\tanh(\kappa (x_{i}^{T}x_{j})+\theta )$

Radial Basis Function (RBF): *K*(*x*_*i*_, *x*_*j*_) = $e^{-\gamma |x_{i}-x_{j}{|}^{2}}$.

The extended version of the two-class SVM that deals with multi-class classification problem by designing a number of one-against-all two-class SVMs [27,36] is used here. For example, a *K*-class problem is handled with *K *two-class SVMs, each of which is used to separate a class of points from all the remaining points.

### Proposed MOGA-SVM Clustering

This section describes the proposed scheme for integrating the multiobjective fuzzy clustering algorithm (MOGA) with the SVM classifier. The combined approach is called MOGA-SVM. The basic observation motivating MOGA-SVM is that if a subset of points are almost always clustered together by most of the non-dominated solutions, then they may safely be considered to be clustered properly. Hence these points may be used for training a classifier, which can thereafter be used for grouping the remaining low confidence points. In MOGA-SVM, all the final non-dominated solutions are given equal importance and a fuzzy majority voting technique is applied to identify the training set. Since SVM is considered one of the best state-of-art classifiers, it is used here for classification. The steps of MOGA-SVM are as follows:

1. Apply MOGA clustering on the given data set to obtain a set *S *= {*s*_1_, *s*_2_, ..., *s*_*N*_}, *N *≤ *P*, (*P *is the population size) of non-dominated solution strings consisting of cluster centers.

2. Using Eq. (2), compute the fuzzy membership matrix *U*^(*i*) ^for each of the non-dominated solutions *s*_*i*_, 1 ≤ *i *≤ *N*.

3. Reorganize the membership matrices to make them consistent with each other, i.e., cluster *j *in the first solution should be equivalent to cluster *j *in all the other solutions. For example, the solution string {(*p*, *q*, *r*), (*a*, *b*, *c*)} is equivalent to {(*a*, *b*, *c*), (*p*, *q*, *r*)}.

4. Mark the points whose maximum membership degree (to cluster *j*, *j *∈ {1, 2, ..., *K*}) is greater than a membership threshold *α *(0 ≤ *α *≤ 1), for at least *βN *solutions, as training points. Here *β *(0 ≤ *β *≤ 1) is the threshold of the fuzzy majority voting. These points are labeled with class *j*.

5. Train the multi-class SVM classifier (i.e., *K *one-against-all two-class SVM classifiers, *K *being the number of clusters) using the selected training points.

6. Predict the class labels for the remaining points (test points) using the trained SVM classifier.

7. Combine the label vectors corresponding to training and testing points to obtain the final clustering for the complete data set.

The sizes of the training and testing sets depend on the two threshold parameters *α *and *β*. Here *α *is the membership threshold, i.e., it is the maximum membership degree above which a point can be considered as a training point. Hence if *α *is increased, the size of the training set will decrease, but the confidence on the training points will increase. On the other hand, if *α *is decreased, the size of the training set will increase but the confidence of the training points will decrease. The parameter *β *determines the minimum number of non-dominated solutions that agree with each other in the fuzzy voting context. If *β *is increased, the size of the training set will decrease but it indicates that more number of non-dominated solutions agree with each other. On the contrary, if *β *is decreased, the size of the training set increases but it indicates a smaller number of non-dominated solutions have agreement among them. Hence both the parameters *α *and *β *are needed to be tuned in such a way so that a tradeoff is achieved between the size and confidence of the training set of SVM. To achieve this, after several experiments, we have set both the parameters to a value of 0.5.

### Data Sets and Preprocessing

#### Yeast Sporulation

This data set [29] consists of 6118 genes measured across 7 time points (0, 0.5, 2, 5, 7, 9 and 11.5 hours) during the sporulation process of budding yeast. The data set is then log-transformed. The Sporulation data set is publicly available at the website . Among the 6118 genes, the genes whose expression levels did not change significantly during the harvesting have been ignored from further analysis. This is determined with a threshold level of 1.6 for the root mean squares of the log2-transformed ratios. The resulting set consists of 474 genes.

#### Yeast Cell Cycle

The Yeast Cell Cycle data set was extracted from a data set that shows the fluctuation of expression levels of approximately 6000 genes over two cell cycles (17 time points). Out of these 6000 genes, 384 genes have been selected to be cell-cycle regulated [37]. This data set is publicly available at the following website: .

#### Arabidopsis Thaliana

This data set consists of expression levels of 138 genes of Arabidopsis Thaliana. It contains expression levels of the genes over 8 time points *viz*., 15 min, 30 min, 60 min, 90 min, 3 hours, 6 hours, 9 hours, and 24 hours [38]. It is available at .

#### Human Fibroblasts Serum

This dataset [39] contains the expression levels of 8613 human genes. The data set has 13 dimensions corresponding to 12 time points (0, 0.25, 0.5, 1, 2, 4, 6, 8, 12, 16, 20 and 24 hours) and one unsynchronized sample. A subset of 517 genes whose expression levels changed substantially across the time points have been chosen. The data is then log2-transformed. This data set can be downloaded from .

#### Rat CNS

The Rat CNS data set has been obtained by reverse transcription-coupled PCR to examine the expression levels of a set of 112 genes during rat central nervous system development over 9 time points [30]. This data set is available at .

All the data sets are normalized so that each row has mean 0 and variance 1.

### Performance Metrics

For evaluating the performance of the clustering algorithms silhouette index [40] is used. Moreover, two cluster visualization tools, namely, Eisen plot and cluster profile plot, have been utilized.

### Silhouette Index

Silhouette index [40] is a cluster validity index that is used to judge the quality of any clustering solution *C*. Suppose *a *represents the average distance of a point from the other points of the cluster to which the point is assigned, and *b *represents the minimum of the average distances of the point from the points of the other clusters. Now the silhouette width s of the point is defined as:

(8) $$s=\frac{b-a}{max\{a,b\}}.$$

silhouette index *s*(*C*) is the average silhouette width of all the data points (genes) and it reflects the compactness and separation of clusters. The value of silhouette index varies from -1 to 1 and higher value indicates better clustering result.

#### Eisen Plot

In Eisen plot [2] (see Fig. 2(a) for an example), the expression value of a gene at a specific time point is represented by coloring the corresponding cell of the data matrix with a color similar to the original color of its spot on the microarray. The shades of red represent higher expression levels, the shades of green represent lower expression levels and the colors towards black represent absence of differential expression. In our representation, the genes are ordered before plotting so that the genes that belong to the same cluster are placed one after another. The cluster boundaries are identified by white colored blank rows.

#### Cluster Profile Plot

The cluster profile plot (see Fig. 2(b) for an example) shows for each cluster the normalized gene expression values (light green) of the genes of that cluster with respect to the time points. Also, the average expression values of the genes of a cluster over different time points are plotted as a black line together with the standard deviation within the cluster at each time point.

### Input Parameters

The values of the different parameters of MOGA and single objective GA are as follows: number of generations = 100, population size = 50, crossover probability = 0.8 and mutation probability = 0.01. Both *α *and *β *are set to 0.5. The parameter values have been set after several experiments. The fuzzy exponent *m *is chosen as in [41,42], and the values of *m *for the data sets Sporulation, Cell Cycle, Arabidopsis, Serum and Rat CNS are obtained as 1.34, 1.14, 1.18, 1.25 and 1.21, respectively. The fuzzy C-means algorithm has been run for 200 iterations unless it converges before that. Each algorithm has been executed for different number of clusters and the solution giving the best silhouette index score is considered.

## Authors' contributions

U. Maulik carried out the literature study and pre-work planning, collected the data sets, developed the code, performed the experiments and prepared the draft of the manuscript. A. Mukhopadhyay did the literaure study, collected the data sets, developed the code, performed the experiments and prepared the draft of the manuscript. S. Bandyopadhyay carried out the pre-work planning, worked on the conceptual part and corrected the draft. All the authors read and approved the final manuscript.
